# Taming Lithium Nucleation and Growth on Cu Current Collector by Electrochemical Activation of ZnF_2_ Layer

**DOI:** 10.1002/advs.202416426

**Published:** 2025-03-26

**Authors:** Viet Phuong Nguyen, Hyung Cheoul Shim, Young‐Woon Byeon, Jae‐Hyun Kim, Seung‐Mo Lee

**Affiliations:** ^1^ Nanomechatronics University of Science and Technology (UST) 217 Gajeong‐ro Daejeon 34113 Republic of Korea; ^2^ Department of Nanomechanics Korea Institute of Machinery & Materials (KIMM) 156 Gajeongbuk‐ro Daejeon 34103 Republic of Korea; ^3^ Advanced Analysis and Data Center Korea Institute of Science and Technology (KIST) Seoul 02792 Republic of Korea; ^4^ School of Materials Science and Semiconductor Engineering University of Ulsan, 93 Daehak‐ro, Nam‐gu Ulsan 44610 Republic of Korea

**Keywords:** current collector, dendrite, LiF‐rich SEI, lithiophilic, lithium‐metal battery

## Abstract

Lithium‐metal anodes are essential for the advancement of next‐generation batteries. However, their practical use is largely hindered by the uncontrollable growth of dendrites and intricate problems associated with fabricating anodes that meet capacity requirements. Here, it is demonstrated that an ultrathin ZnF_2_ layer deposited on the copper foil can produce a novel and efficient current collector to address these challenges. It is observed that ZnF_2_ can be transformed into LiZn alloy and LiF salt in one step by simple electrochemical activation. The resulting LiZn alloy exhibits high lithiophilicity, which reduces overpotential and promotes uniform lithium nucleation, while the LiF salt enhances the solid electrolyte interphase, ensuring uniform lithium growth. This synergistic effect led to a dendrite‐free, densely packed lithium anode with an extended lifespan, achieving over 900 h in symmetric cells at a high current density of 3 mA cm^−2^ and a high cut‐off capacity of 3 mAh cm^−2^. Furthermore, full cells utilizing the lithium anode (Li capacity of 6 mAh cm^−2^) paired with LiNi_0.8_Mn_0.1_Co_0.1_O_2_ cathodes (mass loading of 11.5 mg cm^−2^) demonstrates drastically improved rate capability and excellent cycling stability. This approach holds great promise for developing safer and more efficient lithium‐metal‐based batteries for future energy storage solutions.

## Graphical abstract

Through a single‐step electrochemical activation process, the ultrathin ZnF_2_ layer deposits on a Cu current collector can be transformed into lithiophilic LiZn alloy and LiF salt that can effectively regulate Li nucleation and growth.

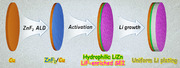

## 1 Introduction

Lithium metal battery that uses metallic lithium (Li) as an anode is likely to be the next generation of energy storage systems.^[^ ^1^ ^]^ Those could include Li‐metal, Li‐sulfur, and Li‐air batteries. To construct these batteries, commercial thick Li foil (over 100 µm, corresponding to a highly overloaded capacity of more than 20 mAh cm^−2^) is typically utilized because it is hard and expensive to reduce the thickness of Li foil to less than 50 µm with current technology.^[^ ^2^, ^3^, ^4^ ^]^ This means that a large excess amount of Li is not utilized, consequently reducing the gravimetric and volumetric capacities significantly. To produce thin Li anodes, roll pressing, molten Li process, and electrodeposition methods are currently being developed.^[^ ^5^, ^6^ ^]^ Among these, electrodeposition can control the thickness of the Li anode more accurately.^[^ ^7^ ^]^ Unfortunately, this method has faced critical challenges related to the morphology of deposited Li and the stability of the solid electrolyte interphase (SEI) layer,^[^ ^7^ ^]^ waiting for an effective technical solution.

Copper (Cu) is widely used as a current collector (CC) and host for Li deposition due to its excellent electrical conductivity and mechanical stability.^[^ ^8^, ^9^, ^10^, ^11^ ^]^ However, its inherent lithiophobicity, characterized by a high nucleation barrier, results in spatially inhomogeneous Li nucleation at the initial deposition stage.^[^ ^12^, ^13^, ^14^, ^15^ ^]^ This non‐uniform nucleation could cause subsequent non‐uniform Li growth. Furthermore, bare Cu cannot facilitate the uniformity of SEI to homogenize the Li‐ion flux.^[^ ^16^ ^]^ Consequently, Li tends to grow on Cu vertically rather than planarly, leading to uncontrollable dendritic Li growth during cycling, limited cycle life, and fast capacity decay of the Li‐metal anode.^[^ ^17^, ^18^, ^19^ ^]^

The stability of Li‐metal anodes is largely determined by two key factors: the Li‐attracting properties (lithiophilicity) of the Cu CC and the durability of the SEI layer.^[^ ^20^, ^21^ ^]^ Researchers have ceaselessly pursued various strategies to improve Li‐metal anode performance.^[^ ^10^, ^20^, ^21^, ^22^, ^23^, ^24^, ^25^, ^26^, ^27^, ^28^ ^]^ These efforts include enhancing the lithiophilicity of Cu CCs, creating artificial SEI layers, modifying electrode/separator interfaces, and developing novel electrolytes and additives. However, these approaches typically address only one aspect of the problem–either improving Cu's lithiophilicity or strengthening the SEI layer–but rarely tackle both issues simultaneously. As a result, they have fallen short of fully stabilizing Li‐metal anodes. Therefore, once a method is developed to simultaneously address both the Li dendrite formation and SEI stability challenges, it would undoubtedly be a technological breakthrough.

Here, we report a facile way to enhance the lithiophilicity of the Cu CC and facilitate the formation of a robust SEI layer in a one‐shot. We observed that the ultrathin ZnF_2_ layer directly deposited on the Cu foil is in situ converted to LiZn alloy and LiF salt during the preliminary discharging process (i.e., (LiF‐LiZn)/Cu). The resulting LiZn alloy showed high lithiophilicity, thereby significantly reducing overpotential and homogenizing Li nucleation. Concurrently, the LiF salt reinforced the SEI to homogenize Li‐ion flux, thereby ensuring the uniformity of Li growth. Thanks to the synergistic effect, a dendrite‐free and dense Li anode with a long lifespan was readily realized. Full cells utilizing our Li anode exhibited significantly enhanced rate capability and cycling stability, showcasing the promising potential of our simple approach in stabilizing the Li anode.

## 2 Preparation of ZnF_2_/Cu and Its Electrochemical Activation

**Figure 1:**
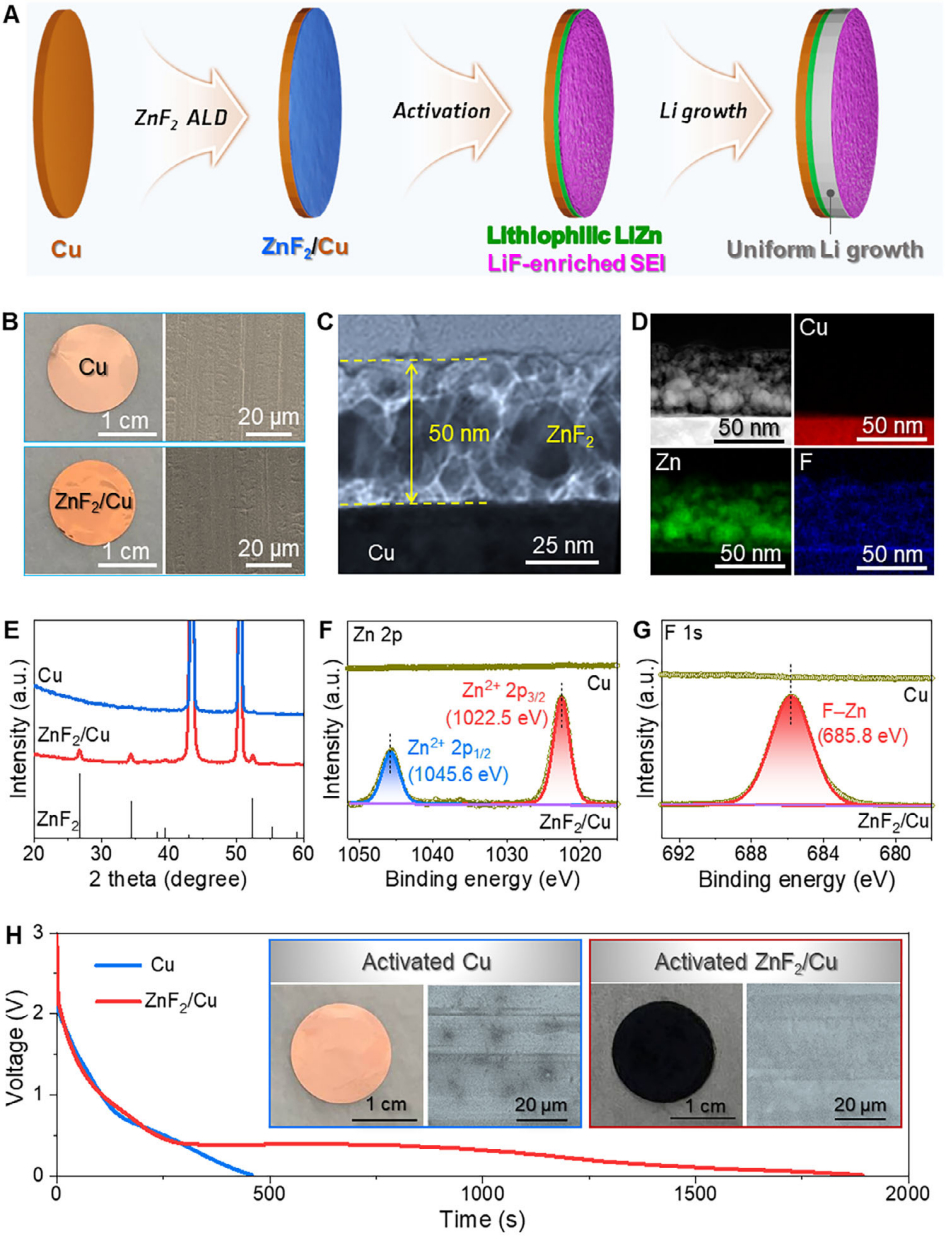
Proposed modification method of Cu current collector. A) Schematic Illustration of uniform Li deposition on Cu current collector triggered by ultrathin ZnF_2_ layer prepared by ALD. Electrochemical activation transformed the ZnF_2_ layer into LiZn alloy and LiF salt, both promoting favorable Li nucleation and growth (See the text for details). B) Optical and SEM images of raw Cu and ZnF_2_ deposited Cu current collector, i.e., ZnF_2_/Cu, respectively. C,D) Bright‐field and dark‐field TEM images along with corresponding EDX mappings of Cu, Zn, and F elements, respectively. E) XRD pattern. F,G) XPS spectra of Zn 2p and F 1s, respectively. H) Discharging profiles of Li║Cu and Li║ZnF_2_/Cu cells to lead to electrochemical activation. The digital photos and SEM images of activated bare Cu and ZnF_2_/Cu current collectors are seen in the insets.

We recognized that the pretreatment of the commercial Cu foil before use is highly needed because the inherent lithiophobic nature of Cu causes uneven Li nucleation, leading to significant growth of Li dendrites (Figure S1, Supporting Information). Motivated by this, we hypothesized that the Li hosting ability of Cu foil could be enhanced by coating it with a suitable material that could simultaneously reduce the nucleation overpotential and regulate Li growth. Based on this hypothesis, we turned our attention to ZnF_2_ because it contains lithiophilic Zn and useful F for LiF formation.^[^ ^21^, ^29^ ^]^ To impeccably deposit a thin and conformal ZnF_2_ layer on Cu foil (i.e., ZnF_2_/Cu), we employed atomic layer deposition (ALD).^[^ ^30^, ^31^ ^]^ We hypothesized that electrochemical activation might be able to allow ZnF_2_ to be transformed into LiZn alloy and LiF salt, respectively, to induce uniform Li nucleation/growth and enhance the stability of the SEI layer (**Figure** **1**A). In the following sections, we will show that our hypothesis was correct.

The ZnF_2_/Cu samples with different ZnF_2_ layer thicknesses were prepared by adjusting the number of ALD cycles. After thorough characterizations, we determined the optimal thickness of the ZnF_2_ layer (≈50 nm by 625 ALD cycles, see the details in the Experimental Section). As shown in Figure 1B, the reddish‐brown color of the Cu foil deepened after ZnF_2_ ALD, while the morphology of the Cu foil remained nearly unchanged. The cross‐section transmission electron microscope (TEM) image exhibited a thin ZnF_2_ layer uniformly covering the Cu foil (Figure 1C; Figure S2, Supporting Information). An interplanar distance of 0.21 Å observed in the high‐resolution TEM (HRTEM) image could be attributed to the (201) plane of ZnF_2_, verifying the formation of ZnF_2_. The energy‐dispersive X‐ray spectroscopy (EDX) analyses indicated a uniform distribution of Zn and F on the Cu surface (Figure 1D; Figure S3, Supporting Information). The crystal structure and the composition of the ZnF_2_ layer were then analyzed by X‐ray diffraction (XRD) and X‐ray photoelectron spectroscopy (XPS). The appearance of the ZnF_2_ peaks in the XRD patterns, and the Zn and F peaks in the XPS spectra confirmed the presence of a ZnF_2_ layer on the Cu foil once more. (Figure 1E; Figure S4, Supporting Information). Notably, the characteristic peak of Cu could not be observed in the XPS survey scan of ZnF_2_/Cu, implying the perfect coverage of the ZnF_2_. Moreover, the difference between the binding energies of Zn 2p_3/2_ (1022.5 eV) and 2p_1/2_ (1045.6 eV) was calculated to be 23.1 eV, consistent with the values for Zn^2+^ (Figure 1F).^[^ ^32^ ^]^ In the F 1s spectrum, the peak centered at 685.8 eV could be assigned to the F─Zn bonds (Figure 1G).^[^ ^33^ ^]^ These results firmly demonstrated that the ultrathin ZnF_2_ layer was successfully deposited on the surface of Cu foil.

To electrochemically activate this ZnF_2_/Cu, we assembled coin cells using ZnF_2_/Cu paired with Li foil. Then, the coin cells were discharged to 0.01 V and operated 5 cycles between 0.01 and 1.0 V (Figure S5, Supporting Information). We observed that unlike the Li║Cu cell showing a rapid voltage drop, the Li║ZnF_2_/Cu cell exhibits a long plateau at 0.4 V, presumably due to the lithiation of ZnF_2_ (Figure 1H). After this operation, we disassembled these cells for further analysis. We observed that the color of the bare Cu electrode remains nearly unchanged, while the ZnF_2_/Cu becomes black (digital photos in Figure 1H). This indicated that noticeable changes likely occur on the surfaces of the ZnF_2_/Cu during this electrochemical reaction. As can be seen in the scanning electron microscope (SEM) images, only a few spots appeared on the activated bare Cu electrode, likely due to the decomposition of the electrolyte. In contrast, the surface of the activated ZnF_2_/Cu was fully and uniformly covered by likely the products of the in situ electrochemical reactions between ZnF_2_ and Li^+^ ions.

**Figure 2:**
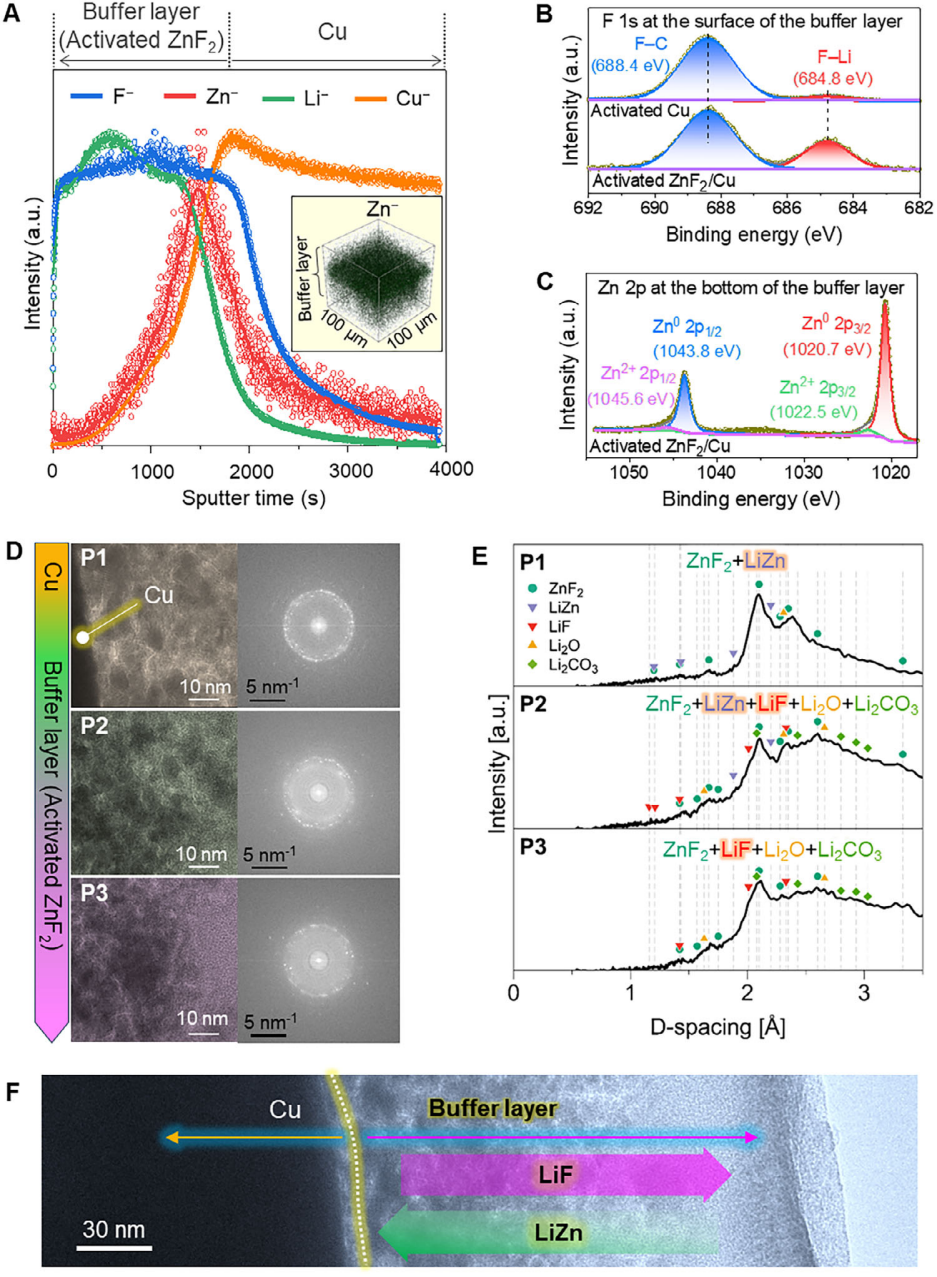
Analysis to monitor the change of ZnF_2_/Cu after electrochemical activation. A) ToF‐SIMS depth profile showing the distribution of F^−^ and Zn^−^ in the electrochemically activated ZnF_2_/Cu. The inset is a 3D reconstruction of Zn^−^ distribution in activated ZnF_2_/Cu. B,C) High‐resolution XPS spectrum of F 1s and Zn 2p after activation, respectively. D) HRTEM images and corresponding SAED patterns measured at different positions (P1, P2, and P3) of the activated ZnF_2_/Cu. E) The plot of D‐spacing against electron diffraction intensity at P1, P2, and P3, respectively. F) HRTEM image of the activated ZnF_2_/Cu and presumable distribution of LiF and LiZn in the buffer layer.

To validate what occurred in the ZnF_2_/Cu during this electrochemical activation, first, the time‐of‐flight secondary‐ion mass spectrometry (ToF‐SIMS) analysis was performed. The results showed that F^−^ is not only distributed in the Zn^−^ area but also evenly spreads throughout the entire buffer layer on the activated ZnF_2_/Cu (**Figure** **2**A). The F^−^ signals likely come from the F‐enriched species formed by the reaction between ZnF_2_ and Li^+^, and from the electrolyte. The XPS depth profiling further identified the compounds containing the F^−^. The F─Li peaks were detected, confirming the formation of LiF salt (Figure 2B). The LiF salts on the activated bare Cu electrode appear to have been generated by the electrolyte decomposition. Meanwhile, the reaction between ZnF_2_ and Li^+^ seemed to have significantly enriched the LiF content in the buffer layer on the activated ZnF_2_/Cu, as evidenced by the strong F─Li peak. The characteristic peaks of Zn 2p were shifted to the lower binding energy compared to the initial values because most Zn^2+^ in ZnF_2_ was reduced to Zn^0^ in LiZn (Figure 2C). TEM analysis provided additional information on the substances generated by the electrochemical activation of ZnF_2_/Cu. Figure 2D,E shows the HRTEM images, selective area electron diffraction (SAED) patterns, and the plot of D‐spacing against electron diffraction intensity measured at different positions of the activated ZnF_2_/Cu, respectively. Near the interface between Cu and ZnF_2_, the signal of LiZn was dominant (P1), and in the intermediate region (P2), both LiZn and LiF were detected. At the end region of the buffer layer (P3), the LiF signal was dominant. These findings were further corroborated by EDX, which detected corresponding Zn and F element signals (Figure S6, Supporting Information). This additional analysis reinforces the observed gradient in composition across the interface. These ToF‐SIMS, XPS, and TEM data indicated that in the buffer layer, the LiF and LiZn are distributed rather unevenly with regional selectivity (Figure 2F). Our analysis strongly indicated that the ultrathin ZnF_2_ on the Cu CC undergoes in situ conversion, forming a (LiF‐LiZn)/Cu composite likely through the following electrochemical reaction: ZnF_2_ + 3Li^+^ + 3e^−^ → LiZn + 2LiF.

## 3 Li Deposition Behavior on the (LiF‐LiZn)/Cu CC

**Figure 3:**
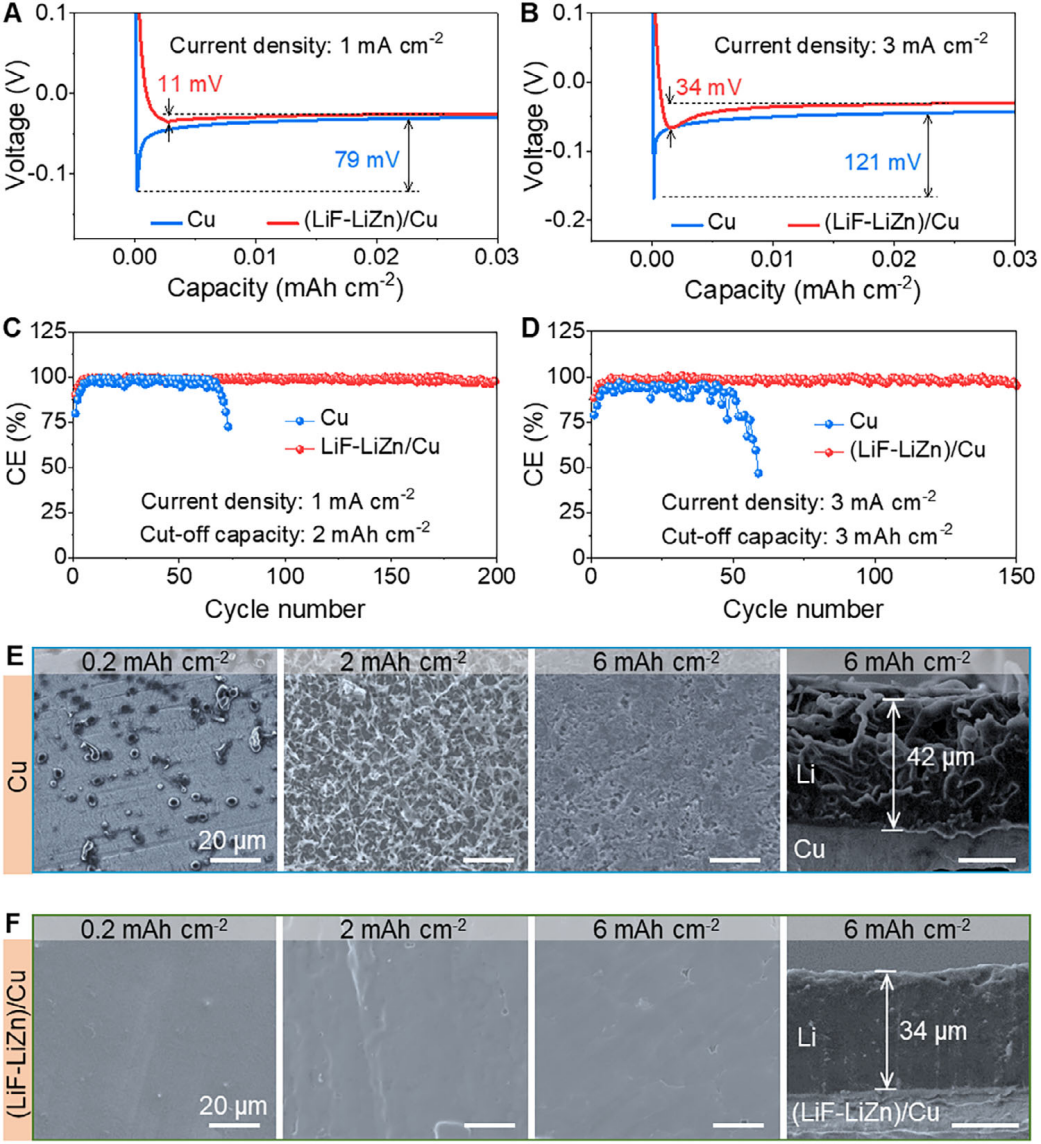
Li deposition on bare Cu and ZnF_2_/Cu current collectors. A,B) Voltage profiles of Li plating showing the nucleation overpotentials at current densities of 1 and 3 mA cm^−2^ on bare Cu and (LiF‐LiZn)/Cu, respectively. C,D) Coulombic efficiency of Li plating/stripping at current densities of 1 and 3 mA cm^−2^ and cut‐off capacities of 2 and 3 mAh cm^−2^, respectively. E,F) Morphologies and thicknesses of Li deposited on bare Cu and (LiF‐LiZn)/Cu, respectively.

The advantages of the in situ formed LiZn alloy and LiF salt in regulating the Li nucleation and growth were evaluated by electrodeposition of Li at various current densities and capacities. As shown in **Figure** **3**A, at a current density of 1 mA cm^−2^, the overpotential of Li nucleation on the (LiF‐LiZn)/Cu CC was recorded as 11 mV. This value remained nearly unchanged even after 200 cycles of Li plating/stripping (Figure S7, Supporting Information) and was significantly lower than that on bare Cu (79 mV). Even when the current density increased to 3 mA cm^−2^, the (LiF‐LiZn)/Cu CC electrode still exhibited a lower overpotential (Figure 3B). The significant reduction of the overpotential could be attributed to the improved lithiophilicity of the (LiF‐LiZn)/Cu CC.^[^ ^31^, ^34^ ^]^ Indeed, the results of theoretical calculations showed that the adsorption energy of Li on LiZn alloy was considerably higher than that on bare Cu (Figure S8, Supporting Information), testifying to the high affinity between Li^+^ ions and LiZn alloy.^[^ ^35^ ^]^ Furthermore, we observed that on the bare Cu CC, Li plating/stripping lasted only 65 cycles, likely due to the formation of Li dendrites, which eventually transformed into inactive Li (Figure S9, Supporting Information).^[^ ^36^, ^37^ ^]^ In contrast, the (LiF‐LiZn)/Cu CC could enable stable Li plating/stripping with the average Coulombic efficiency (CE) of 99.2% for more than 200 cycles at 1 mA cm^−2^ with a cut‐off capacity of 2 mAh cm^−2^ (Figure 3C). The initial CE of Li plating/stripping on the (LiF‐LiZn)/Cu CC was also found to be improved, indicating enhanced reversibility. When the current density and cut‐off capacity increased to 3 mA cm^−2^ and 3 mAh cm^−2^, respectively, the CE was as high as 98.4% for more than 150 cycles (Figure 3D). Even at a higher current density of 5 mA cm^−2^, (LiF‐LiZn)/Cu demonstrated excellent reversibility of Li plating/stripping (Figure S10, Supporting Information). The voltage hysteresis of the Li plating/stripping on the (LiF‐LiZn)/Cu CC was found to be greatly reduced (Figures S11 and S12, Supporting Information). The roles of the in situ formed LiZn alloy and LiF‐enriched SEI in regulating the Li nucleation and growth were further disclosed by the morphologies of Li deposition with different capacities at a current density of 1 mA cm^−2^. The SEM images in Figure 3E showed that Li nuclei on the lithiophobic Cu CC surface are unevenly distributed. Due to the tip effect, Li^+^ ions likely tended to deposit at the exiting Li nuclei.^[^ ^38^ ^]^ Further, Li deposition led to the formation of elongated and whisker‐like dendrites and loose structures with numerous voids. At a capacity of 6 mAh cm^−2^, the thickness of Li deposited on the bare Cu CC was measured to be ≈42 µm. Meanwhile, the morphology of Li deposited on the (LiF‐LiZn)/Cu CC was completely different (Figure 3F). Thanks to the uniform distribution of highly lithiophilic LiZn alloy that acted as preferential nucleation seeds, Li nuclei likely formed in the form of the film without random agglomeration. Subsequently, the LiF‐enriched SEI regulated the Li‐ion flux, resulting in homogeneous and dense Li growth. Therefore, at a capacity of 6 mAh cm^−2^, Li deposited on the (LiF‐LiZn)/Cu CC had a thickness of just ≈34 µm, which was much smaller than that on the bare Cu CC. Furthermore, upon complete Li stripping, the (LiF‐LiZn)/Cu CC surface maintained pristine cleanliness, whereas random Li agglomeration persisted on the bare Cu CC (Figure S13, Supporting Information). So far, we have provided a clear mechanistic understanding of how pre‐deposited metal fluorides are transformed and subsequently influence Li deposition, offering new insights into interfacial engineering for next‐generation Li‐metal anodes.

## 4 Probing the Cycling Stability

**Figure 4:**
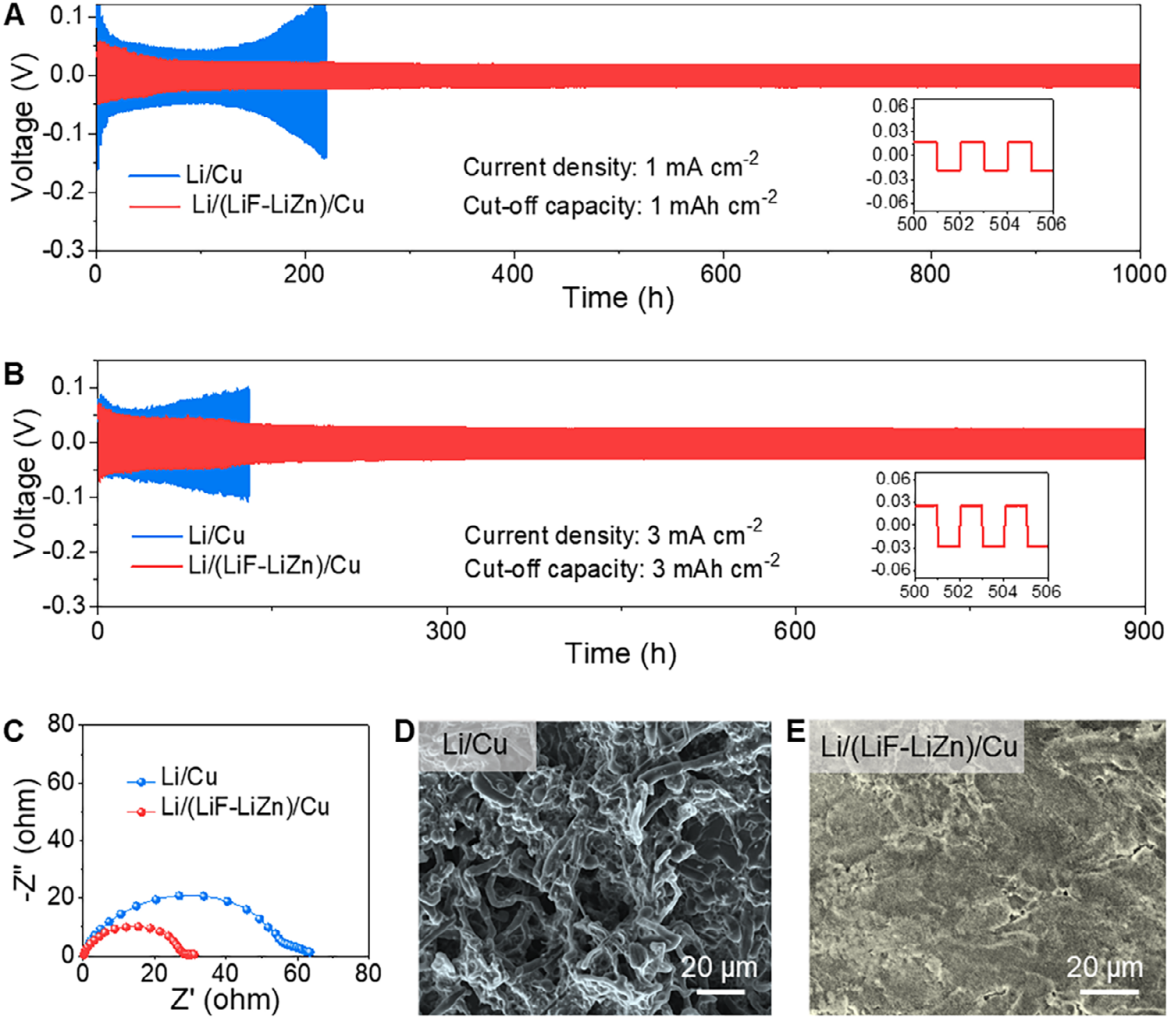
Stability of the Li‐metal anode. A,B) Long‐term cycling performance of symmetric cells at current densities of 1 and 3 mA cm^−2^ and cut‐off capacities of 1 and 3 mAh cm^−2^, respectively. C) EIS plots of symmetric cells after 10 cycles. D,E) SEM images Li/Cu and Li/(LiF‐LiZn)/Cu, respectively, after the cycling test.

The cycling stability of the Li/(LiF‐LiZn)/Cu electrode (Figure 3F, Li capacity of 6 mAh cm^−2^) was investigated using symmetric cells and compared with that of the Li/Cu counterpart (Figure 3E). The symmetric cells with the Li/(LiF‐LiZn)/Cu electrodes exhibited steady cyclability over 1000 h with a small voltage hysteresis of 17 mV at the 1 mA cm^−2^ and 1 mAh cm^−2^ (**Figure** **4**A). In sharp contrast, the symmetric cells with the Li/Cu electrode could work for a short time due to a gradual increase in voltage hysteresis. Astonishingly, when the current density and the cut‐off capacity were adjusted up to 3 mA cm^−2^ and 3 mAh cm^−2^, the excellent stability of the Li/(LiF‐LiZn)/Cu electrode was well maintained, showing the promise for the application in fast‐charging Li‐metal battery. The Li/(LiF‐LiZn)/Cu electrode could stably cycle with a voltage hysteresis of ≈25 mV, while the other exhibited seriously reduced cycle life (Figure 4B). Interestingly, the symmetric cells with the Li/(LiF‐LiZn)/Cu electrodes were still able to work well at extremely high current densities of 5 and 10 mA cm^−2^ (Figure S14, Supporting Information). The cycling stability of the Li/(LiF‐LiZn)/Cu electrode was comparable to or even outperformed previously reported data (Table S1, Supporting Information). Electrochemical impedance spectroscopy (EIS) was conducted to examine the interface resistance of the electrodes. The cell using Li/(LiF‐LiZn)/Cu electrodes displayed a much lower charge transfer resistance than the one using Li/Cu electrodes (Figure 4C). The largely reduced charge transfer resistance of the cell using Li/(LiF‐LiZn)/Cu electrodes could be attributed to the LiF‐enriched SEI layer. To better understand the electrochemical stability, the morphology evolution of these electrodes was further investigated. As shown in Figure 4D, the Li/Cu electrode presented a rough surface entirely enveloped by irregular, whisker‐shaped Li dendrites, accompanied by extensive gaps that exposed a loose and porous structure. These Li dendrites substantially augmented the surface area of deposited Li metal, causing continuous electrolyte consumption to form passivation layers and decayed cyclability. However, in the case of the Li/(LiF‐LiZn)/Cu electrode, a clean surface without Li dendrites and dense structure was well preserved (Figure 4E), explaining its excellent cycling stability. Given the capability of working at high current densities and long‐term cycling performance, Li/(LiF‐LiZn)/Cu could be regarded as an appealing anode for LMB applications.

## 5 Full Battery Performance

**Figure 5:**
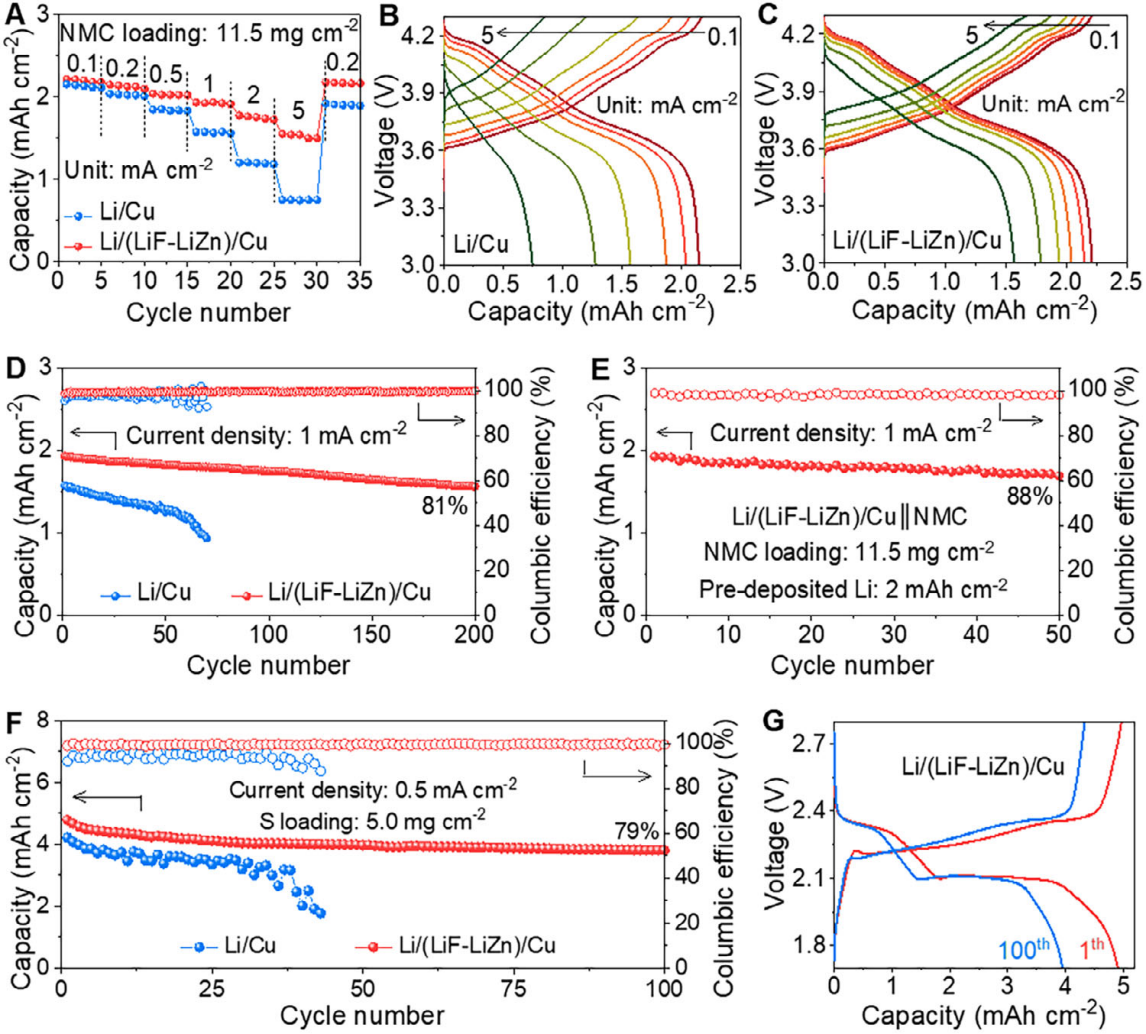
Electrochemical performance of the Li‐metal full cells. A) Rate capability of different Li║NMC full cells. B,C) Discharge and charge voltage profiles of Li/Cu║NMC and Li/(LiF‐LiZn)/Cu║NMC full cells, respectively, at various current densities. D) Long‐term cycling performance of different Li║NMC full cells. E) Cycling performance of the Li/(LiF‐LiZn)/Cu║NMC “anode‐less” cell with the N/P ratio of 0.9. F) Long‐term cycling performance of the Li/(LiF‐LiZn)/Cu║S and Li/Cu║S full cells. G) Discharge and charge voltage profiles of the Li/(LiF‐LiZn)/Cu║S full cell at the 1st and 100th cycle.

We probed the electrochemical performance of the high‐voltage full‐cell composed of Li/(LiF‐LiZn)/Cu as an anode and a LiNi_0.8_Mn_0.1_Co_0.1_O_2_ (NMC) as a cathode (i.e., Li/(LiF‐LiZn)/Cu║NMC cell). The Li/Cu║NMC cell was also assembled for comparative study. The mass loading of NMC was 11.5 mg cm^−2^, and the capacity of pre‐deposited Li was 6 mAh cm^−2^. The negative‐to‐positive (N/P) capacity ratio was calculated to be 2.7. The rate capability of these two cells is shown in **Figure** **5**A. At a low current density of 0.1 mA cm^−2^, both two cells delivered similar capacities. However, as the current density increases, the Li/(LiF‐LiZn)/Cu║NMC cell exhibited significantly higher capacities than the Li/Cu║NMC cell. At a high current density of 5 mA cm^−2^, the Li/(LiF‐LiZn)/Cu║NMC cell still delivered a high discharge capacity of 1.55 mAh cm^−2^, while the capacity of the Li/Cu║NMC cell quickly dropped to 0.73 mAh cm^−2^. The enhanced rate capability of the Li/(LiF‐LiZn)/Cu║NMC cell could be attributed to the superior kinetic properties of the Li/(LiF‐LiZn)/Cu anode, particularly when operating at high current densities. In addition, the charge and discharge plateau of the Li/Cu║NMC cell almost disappeared, accompanied by a large hysteresis voltage (Figure 5B). In contrast, the Li/(LiF‐LiZn)/Cu║NMC cell well maintained the characteristic shape of the charge–discharge curves (Figure 5C) and showed better cycling stability compared to the Li/Cu║NMC cell (Figure 5D; Figure S15, Supporting Information). After 200 cycles at 1 mA cm^−2^, the capacity retention of the Li/(LiF‐LiZn)/Cu║NMC cell was calculated to be 81%, along with well‐maintained charge–discharge plateaus during repeated cycling. Conversely, the Li/Cu║NMC cell operated only ≈60 cycles before a rapid capacity drop. Even more encouragingly, the “anode‐less” cell utilizing the Li/(LiF‐LiZn)/Cu anode, with only 2 mAh cm^−2^ of pre‐deposited Li (N/P ratio reduced to 0.9), operated stably, achieving capacity retention of 88% after 50 cycles (Figure 5E; Figure S16, Supporting Information). This result indicated the excellent potential of Li/(LiF‐LiZn)/Cu for enabling high‐performance “anode‐less” Li batteries.

The Li anode is also known to be a critical component that directly affects the performance of lithium‐sulfur batteries.^[^ ^39^, ^40^, ^41^ ^]^ Therefore, to further verify the applicability of the (LiF‐LiZn)/Cu CC, we also assembled a cell using the Li/(LiF‐LiZn)/Cu as an anode and a high‐capacity S as a cathode. To restrain the shuttle effect of lithium polysulfides and promote conversion reactions, we employed MXene‐driven TiO_2_/TiS_2_ as a sulfur host (Figure S17, Supporting Information), whose high performance had been already proven in the previous study.^[^ ^42^ ^]^ The mass loading of S was as high as 5.0 mg cm^−2^, and the capacity of pre‐deposited Li was 6 mAh cm^−2^. The lithium‐sulfur battery full cell initially reached a high capacity of 4.79 mAh cm^−2^. Because of the high stability of both the anode and cathode, the discharge capacity of the cell using the Li/(LiF‐LiZn)/Cu anode was recorded to be up to 3.80 mAh cm^−2^ together with a capacity retention of 79% (Figure 5F,G). These results confirmed that our current collector could enable the enhanced rate capability and cycling stability of not only high‐voltage full cells using intercalation cathodes but also high‐capacity full cells using conversion cathodes.

## 6 Conclusion

In summary, we demonstrated that the ultrathin ZnF_2_ layer deposited on the Cu current collector can be converted into LiZn alloy and LiF salt in a single step by simple electrochemical activation (ZnF_2_ + 3Li^+^ + 3e^−^ → LiZn + 2LiF). From these reactions, the raw Cu foil was directly transformed into a new type of current collector in the form of (LiF‐LiZn)/Cu. The LiZn alloy significantly reduced overpotential and promoted uniform Li nucleation due to its remarkable lipophilicity. Meanwhile, the LiF salt reinforced the SEI, regulating Li^+^ flux and ensuring uniform Li growth. The synergistic effects created by LiZn and LiF effectively prevented the formation of unwanted Li dendrites while stabilizing the Li anode structure during electrochemical cycling. Consequently, the newly developed Li/(LiF‐LiZn)/Cu anode exhibited exceptional rate capability and cycling stability, showcasing superior performance in both high‐voltage and high‐capacity cells. This simple and feasible strategy is expected to offer great potential for the advancement of next‐generation Li‐metal‐based battery systems.

## Conflict of Interest

The authors declare no conflicts of interest.

## Supporting information



Supporting Information

## Data Availability

The data that support the findings of this study are available from the corresponding author upon reasonable request.
