# Supplementary material for: Taming Lithium Nucleation and Growth on Cu Current Collector by Electrochemical Activation of ZnF2 Layer
Source: Adv Sci (Weinh). 2025 Mar 26;12(19):2416426. doi: 10.1002/advs.202416426 (PMC12097123; doi:10.1002/advs.202416426)
Supplement: Supplementary file 1 — Supporting Information [file ADVS-12-2416426-s001.pdf]

ADVANCED SCIENCE
Open Access

Supporting Information

for *Adv. Sci.*, DOI 10.1002/advs.202416426

Taming Lithium Nucleation and Growth on Cu Current Collector by Electrochemical Activation of ZnF_2 Layer

*Viet Phuong Nguyen, Hyung Cheoul Shim, Young-Woon Byeon, Jae-Hyun Kim and Seung-Mo Lee**

SUPPORTING INFORMATION

Taming Lithium Nucleation and Growth on Cu Current Collector by Electrochemical Activation of ZnF_2 Layer

Viet Phuong Nguyen,[a,b] Hyung Cheoul Shim,[a,b] Young-Woon Byeon,[c] Jae-Hyun Kim,[a,b] Seung-Mo Lee[a,b,*]

[a] Nanomechatronics, University of Science and Technology (UST), 217 Gajeong-ro, Daejeon 34113, Republic of Korea

[b] Department of Nanomechanics, Korea Institute of Machinery & Materials (KIMM), 156 Gajeongbuk-ro, Daejeon 34103, Republic of Korea

[c] Advanced Analysis and Data Center, Korea Institute of Science and Technology (KIST), Seoul 02792, Republic of Korea

* Corresponding author: sm.lee@kimm.re.kr

1. Experimental section

Preparation of ZnF_2/Cu

The ultrathin ZnF_2 layer was rapidly prepared using atomic layer deposition (ALD). Cu foil was cleaned with ethanol and then loaded into an ALD chamber (S200, Savannah, Cambridge NanoTech Inc.). The ZnF_2 deposition was performed using diethylzinc ($Zn(C_2H_5)_2$, DEZ, Sigma-Aldrich) and HF−pyridine solution (70 wt % HF, Sigma-Aldrich) as precursors. The ALD process condition was optimized and set in the exposure mode with 0.01 s pulse, 20 s exposure, and 30 s purging of DEZ, followed by 1 s pulse, 20 s exposure, and 30 s purging of HF−pyridine for each ALD cycle at a constant N_2 flow rate of 20 sccm. The process temperature and pressure were set at 150 °C and ~0.1 Torr, respectively. The ALD process was conducted for various cycle numbers and optimized at 625 cycles.

Preparation of Li/(LiF-LiZn)/Cu

Li/(LiF-LiZn)/Cu was prepared by electrodeposition of Li on ZnF_2/Cu. The ZnF_2/Cu and Li foil were installed as electrodes into 2032-type coin cells. The electrolyte was composed of 1 M lithium bis(trifluoromethanesulfonyl)imide (LiTFSI) in a mixture of 1,3-dioxolane (DOL) and 1,2-dimethoxyethane (DME) (v/v = 1:1) with 1 wt.% $LiNO_3$ additive. Celgard 2500 was used as the separator. All cells were first discharged to 0.01 V to activate the ZnF_2 layer, followed by repeatedly cycled (5 times) between 0.01 – 1 V at a current density of 0.01 mA cm^{-2} to stabilize the SEI layer before depositing Li. Finally, constant current densities of 1 and 3 mA cm^{-2} were applied for electroplating lithium to obtain Li/(LiF-LiZn)/Cu.

Material characterization

The analysis of the morphology and the element was carried out using FE-SEM (JEOL, JSM-7800F) and EDX (Oxford AZtec® EDX system), respectively. XRD spectrum was collected using an Empyrean diffractometer (PANalytical with Cu Kα (λ = 1.5418 Å)). The microstructure and chemical compositions of the electrode materials were investigated using transmission electron microscopy (TEM) with Talos G1 (Thermo Fisher Scientific) at 200 kV. To prevent air exposure, all sample preparation processes were conducted by utilizing an argon-filled glove box and the air-tight sample transfer systems: Gatan Alto2500 for FIB, and Gatan Model 648 holder for TEM. TEM samples were prepared using a dual-beam FIB (Quanta 3D FEG, Thermo Fisher Scientific) at –170°C with an epoxy protection layer on the surface. Selected area electron diffraction (SAED) patterns were acquired using a 100 nm diameter aperture. The SAED patterns from different regions (P1-P3 in Figure 2) were processed by first obtaining the integrated radial intensity profile, followed by background subtraction using a power-law fit. Subsequently, the inverse of the x-values was taken to convert the radius in the FFT space (1/nm) to D-spacing (Å), and the intensity distribution of the diffraction spots was plotted as a function of D-spacing. Energy-dispersive X-ray spectroscopy (EDS) was performed using a Super-X windowless detector (Thermo Fisher Scientific) with a solid angle of 0.7 srad. Each

EDS elemental mapping was recorded for 4 minutes with an electron dose rate of 39.3 $e^{-}/Å^{2}\cdot s$. We confirmed there is no significant damage after the electron dose for EDS mapping.

A ToF-SIMS spectrometer (TOF-SIMS5, ION-TOF GmbH) was used to perform depth profiling of the samples. A 30 keV primary-ion gun was employed for elemental analysis, utilizing a Bi^{3+} primary-ion beam scanned over an area of 100 × 100 μm^2. Depth profiling was conducted using a 0.5 keV O_2 sputter beam over an area of 300 × 300 μm^2. The lithiated samples were disassembled in an argon-filled glove box and then transferred to the ToF-SIMS using a custom-designed transfer holder, ensuring negligible air exposure.

X-ray photoelectron spectroscopy (XPS) depth profile analysis was performed using a Nexsa G2 spectrometer (Thermo Scientific). The lithiated samples were dried in an argon-filled glove box after careful washing with dimethyl carbonate (DMC). During the transfer of the lithiated samples, they were sealed in an air-tight holder to prevent air exposure. An anode source at 15 kV was used for the measurements, conducted under a vacuum level of 10^{-8} Torr. Survey scans were performed with a step size of 1.0 eV, followed by high-resolution scans with a step size of 0.05 eV. For the sputter phase, a 2 kV monatomic Ar^{+} ion beam was used, with a sputter raster size of 2 mm × 2 mm.

Li‖Li symmetric cell assembly and electrochemical measurements

Li/(LiF-LiZn)/Cu disks with a diameter of 16 mm were used as both electrodes in symmetric cells for cycling stability tests. The electrolyte was composed of 1 M LiTFSI in a mixture of DOL and DME (v/v = 1:1) with 1 wt.% $LiNO_3$ additive. The symmetric cells were repeatedly cycled under the constant current densities of 1 and 3 mA cm^{-2} and the capacities of 1 and 3 mAh cm^{-2}, respectively.

Li‖NMC811 full cell assembly and electrochemical measurements

The cathode was prepared by homogeneously mixing commercial MNC811 (Sigma-Aldrich) with conductive carbon and polyvinylidene fluoride (PVDF) binder with a ratio of 9:0.5:0.5 by weight in N-methyl-2-pyrrolidinone (NMP). The resulting slurry was then coated on a piece of aluminum foil with the active-material loading controlled at 11.5 mg cm^{-2} and dried at 70 °C for 24 h under a vacuum. The composite foil was punched into disks with a diameter of 14 mm and used as the cathode. The as-prepared Li/Cu or Li/(LiF-LiZn)/Cu with pre-deposited Li of 2 or 6 mAh cm^{-2} was utilized as the anode. The electrolyte was 1 M $LiPF_6$ in a mixed solution composed of ethylene carbonate (EC), dimethyl carbonate (DMC), and ethyl methyl carbonate (EMC) (v/v/v=1:1:1), and the separator was Celgard 2500. The carbonate electrolyte was used to enable the utilization of NCM cathodes, owing to their high oxidation stability. The electrochemical performance of the cells was carried out using a Wonatech battery cycler between 2.8 and 4.2 V at room temperature.

Li‖S full cell assembly and electrochemical measurements

The S cathode was composed of S and TiO_2/TiS_2 as the S host. The TiO_2/TiS_2 material was prepared by the entire sulfurization of the $Ti_3C_2T_x$ MXene. Typically, commercial multilayer $Ti_3C_2T_x$ MXene (purchased from Invisible, South Korea) and S powder with a mass ratio of 1:2 were loaded into a quartz glass tube which was prepared by sealing one end of a Pasteur pipette (120 mm length, 8 mm diameter). After that, the tube was evacuated and sealed. The sealed tube was sintered in a furnace at 400 °C for 1 h. After cooling down to room temperature, the tube was opened and then annealed in the furnace at 500 °C for another 1 h to remove excessive S. The obtained TiO_2/TiS_2 material was mixed with sulfur powder with a weight ratio of 3:7. Then, the mixtures were transferred to an autoclave and heated at 156 °C for 12 h to obtain the $S/TiO_2/TiS_2$. After that, the $S/TiO_2/TiS_2$ composite was mixed with conductive carbon and PVDF binder with a ratio of 9:0.5:0.5 in NMP. The resulting slurry was then cast on a piece of aluminum foil with the active-material loading controlled at 5.5 mg cm^{-2} and dried at 70 °C for 24 h under a vacuum. The coated foil was punched into disks with a diameter of

14 mm and used as the cathode. The as-prepared Li/Cu or Li/(LiF-LiZn)/Cu with pre-deposited Li of 6 mAh cm^{-2} was utilized as the anode. The electrolyte was composed of 1 M LiTFSI in a mixture of DOL and DME (v/v = 1:1) with 1 wt.% $LiNO_3$ additive and the separator was Celgard 2500. The electrochemical performance of the cells was carried out using a Wonatech battery cycler between 1.7 and 2.8 V at room temperature.

Theoretical calculation

All *ab initio* calculations were performed using the density functional theory (DFT) method with the Quantum ESPRESSO package. The generalized gradient approximation (GGA) exchange-correlation functional proposed by Perdew, Burke, and Ernzerhof (PBE) was applied. The core electrons were described by ultrasoft pseudopotentials. The popular DFT-D3 method was used to introduce dispersion corrections. Electronic wave functions and the charge density were expanded in a plane-wave basis set using energy cutoffs of 50 and 450 Ry, respectively. Into the supercell, a vacuum layer of 15 Å was inserted to avoid the overlap between different surface supercells. The criterion of convergence was set for residual forces of less than 0.002 Ry $Å^{-1}$ and a change in the total energy of less than 1×10^{-6} Ry. The visualization models were constructed using VESTA software.

The binding energy (E_b) between Cu or LiZn and a Li atom was calculated using

$$E_b = E_{total} - E_{sub} - E_{Li} \quad (1)$$

where E_{total} represents the energy of Cu or LiZn bound with a Li atom, E_{sub} is the energy of the Cu or LiZn substrate, and E_{Li} is the energy of the Li atom in a vacuum.

2. Supporting figures

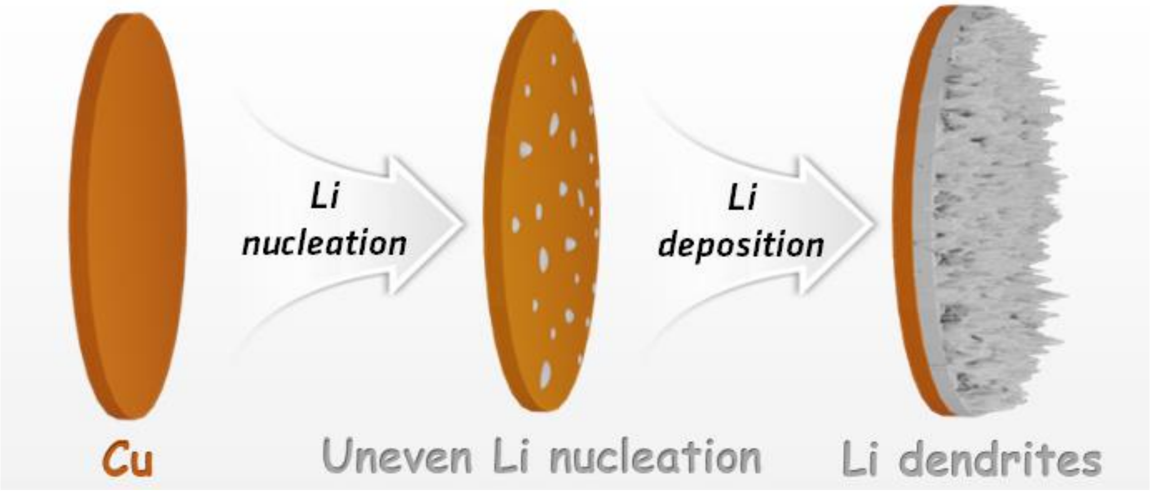

Figure S1. Uneven Li nucleation and growth on lithiophobic Cu foil.

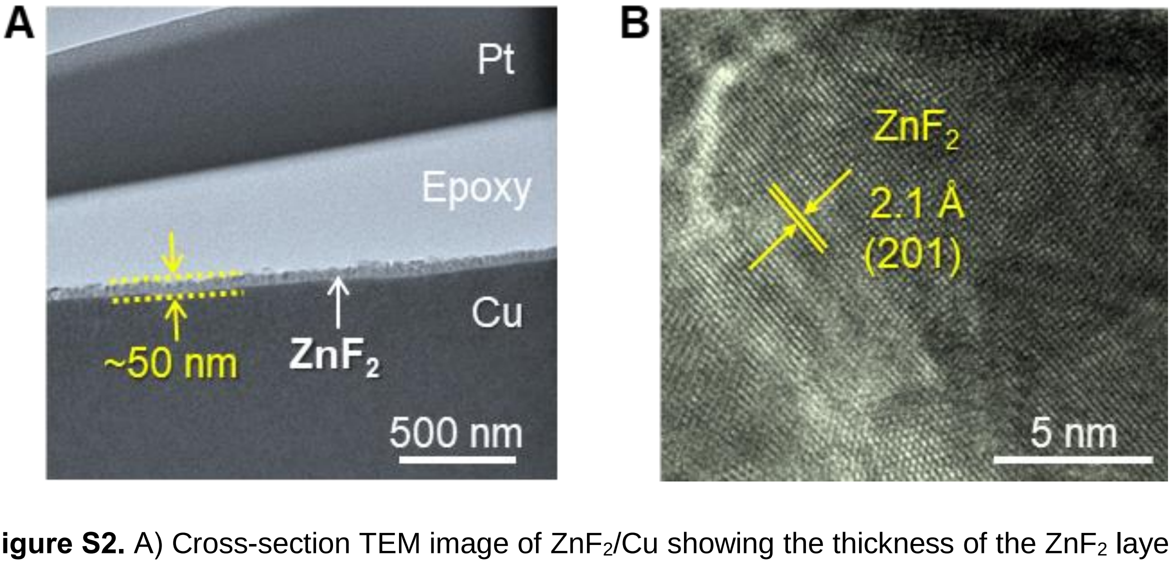

Figure S2. A) Cross-section TEM image of ZnF_2/Cu showing the thickness of the ZnF_2 layer. B) High-resolution TEM image of the ZnF_2 layer.

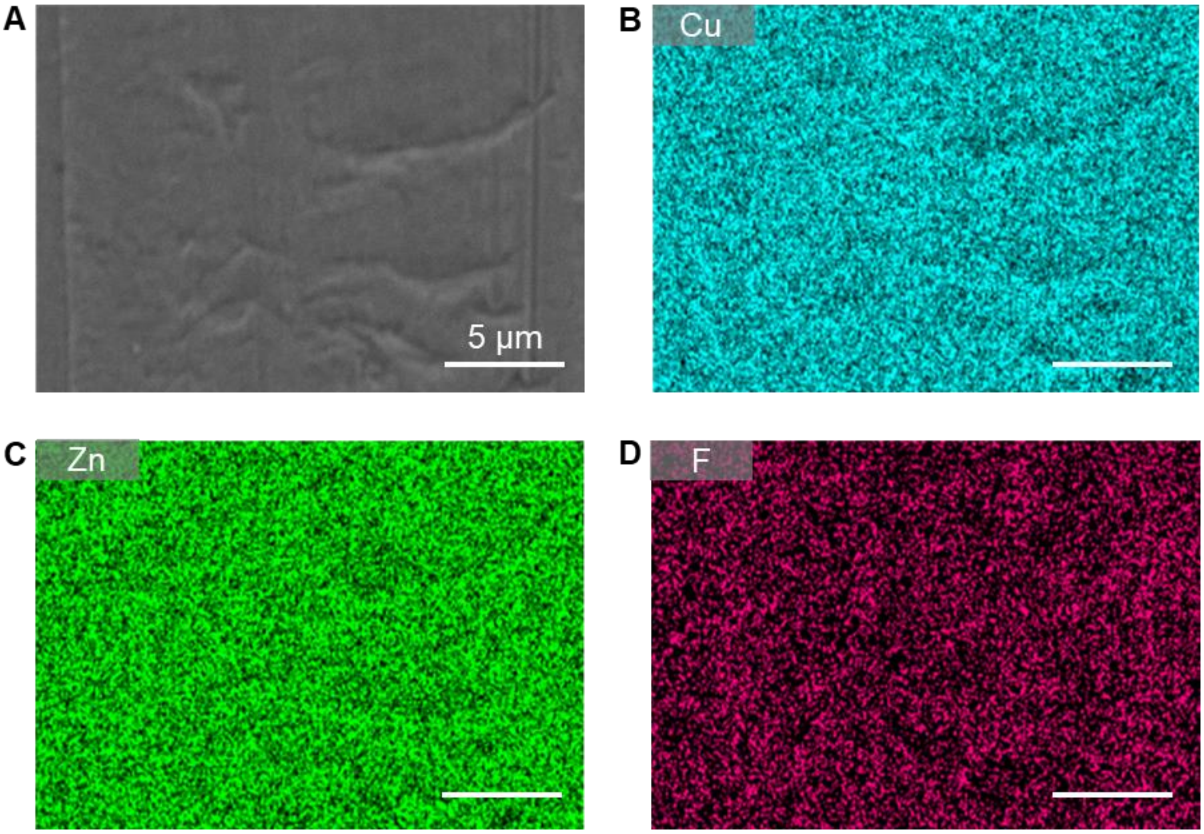

Figure S3. A) SEM image of ZnF_2/Cu. B–D) Corresponding mappings of Cu, Zn, and F elements, respectively.

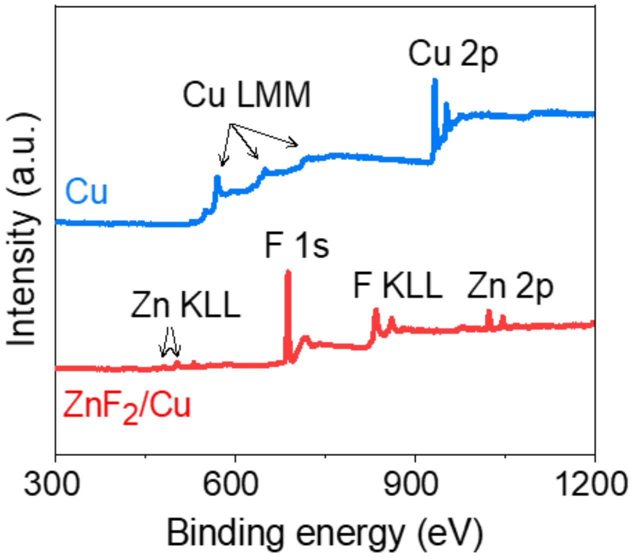

Figure S4. XPS survey of Cu and ZnF_2/Cu.

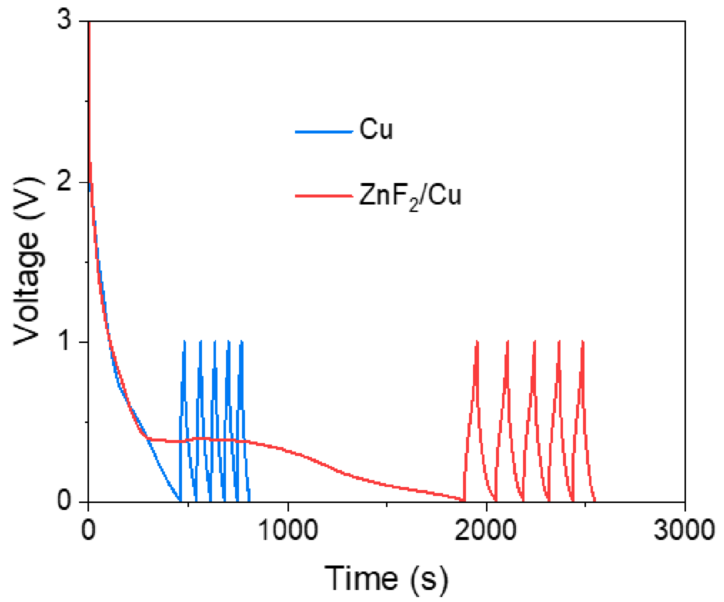

Figure S5. Voltage profiles of Li‖Cu and Li‖ZnF_2/Cu cells in the activation and stabilization processes.

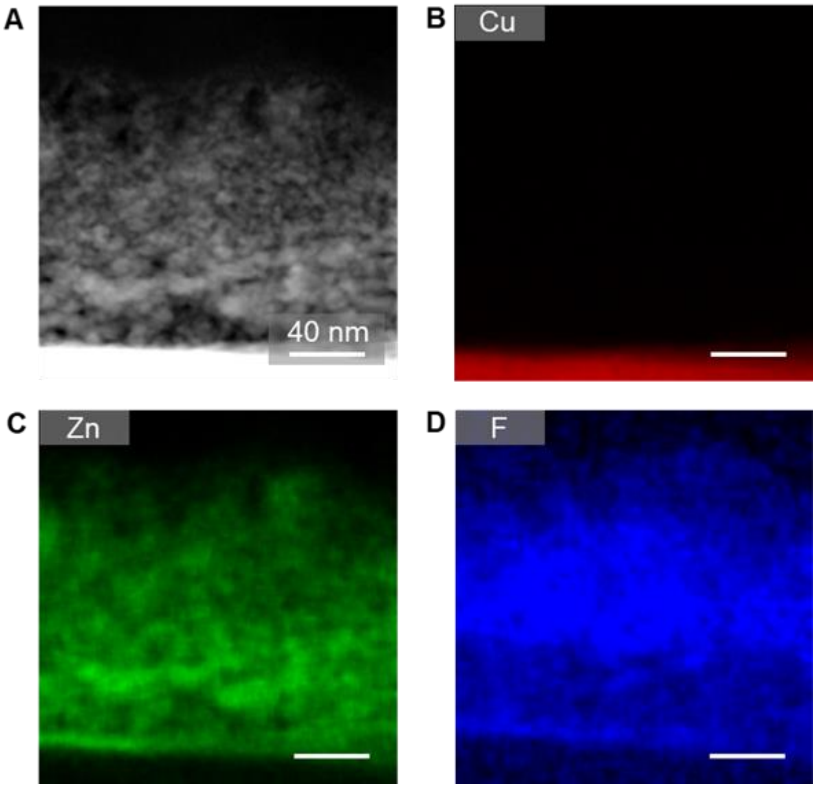

Figure S6. A) High-angle annular dark-field TEM image of activated ZnF_2/Cu. B–D) Corresponding EDX mapping images of the Cu, Zn, and F elements, respectively.

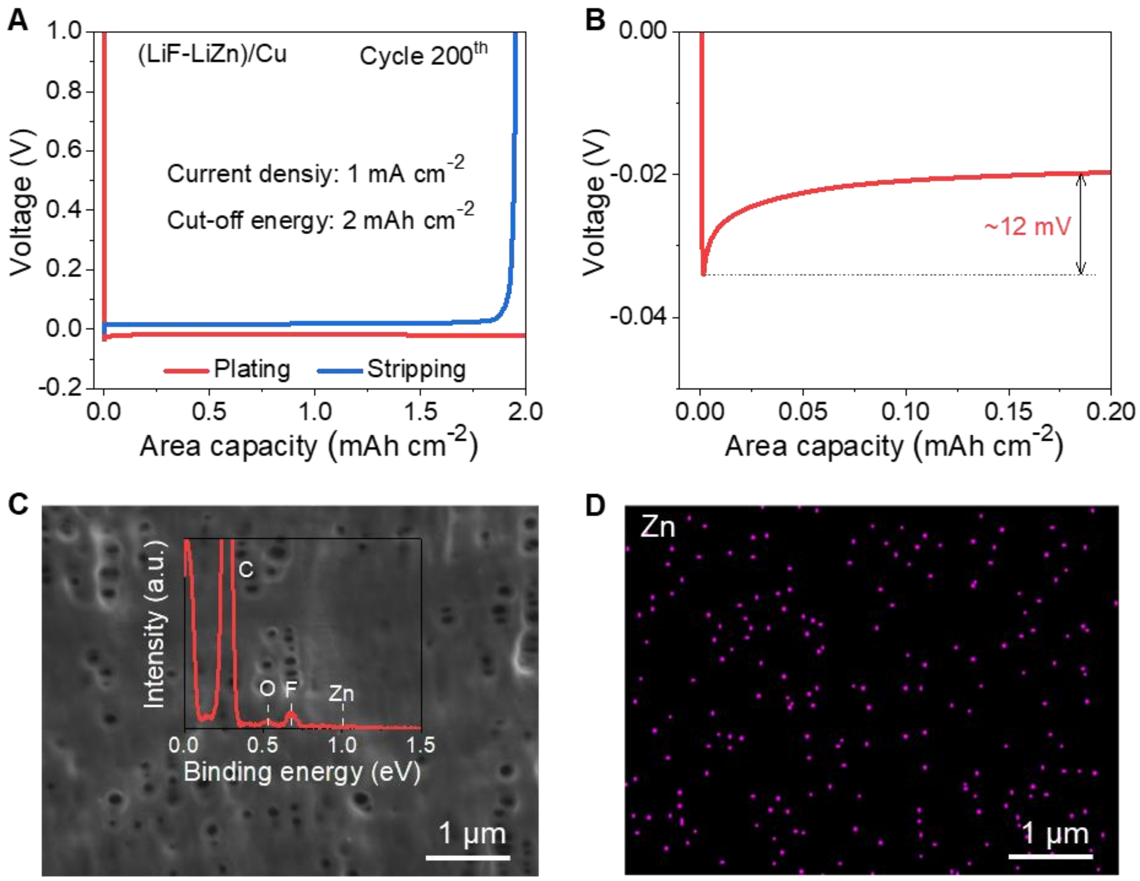

Figure S7. A–B) Voltage profiles and corresponding nucleation overpotential for Li plating and stripping on the (LiF-LiZn)/Cu current collector at 1 mA cm^{-2}, measured during the cycle 200th cycle. C–D) SEM image of the separator after the plating/stripping test, along with the corresponding Zn distribution map. Even after a long-term plating/stripping test, the overpotential of Li nucleation remained nearly constant. The EDX analysis of the separator showed no significant Zn signal. The absence of detectable Zn on the separator indicates that Zn is not migrating away from the electrode surface.

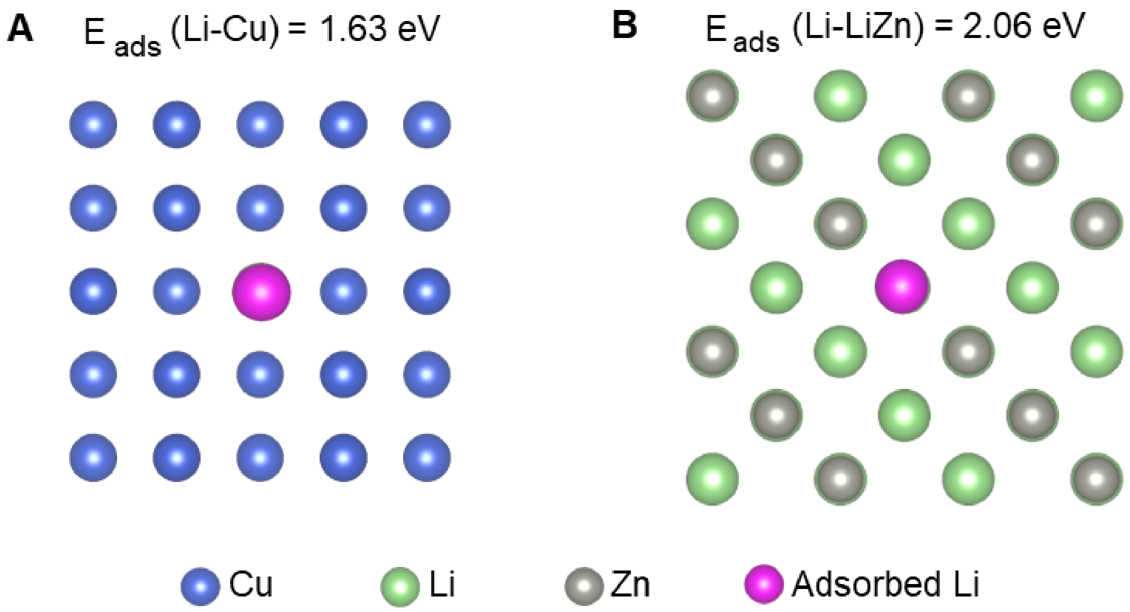

Figure S8. Optimized configurations of Li atom adsorbed on (A) bare Cu and (B) LiZn alloy.

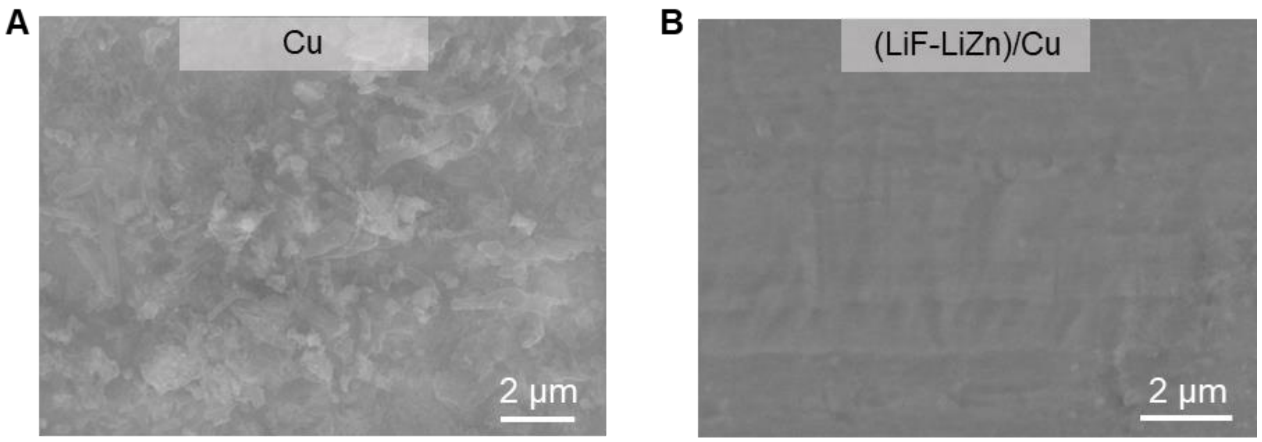

Figure S9. SEM images showing the morphologies of the bare Cu and (LiF-LiZn)/Cu CCs after the plating/stripping test, respectively. Nearly no dead Li was observed on the (LiF-LiZn)/Cu CC.

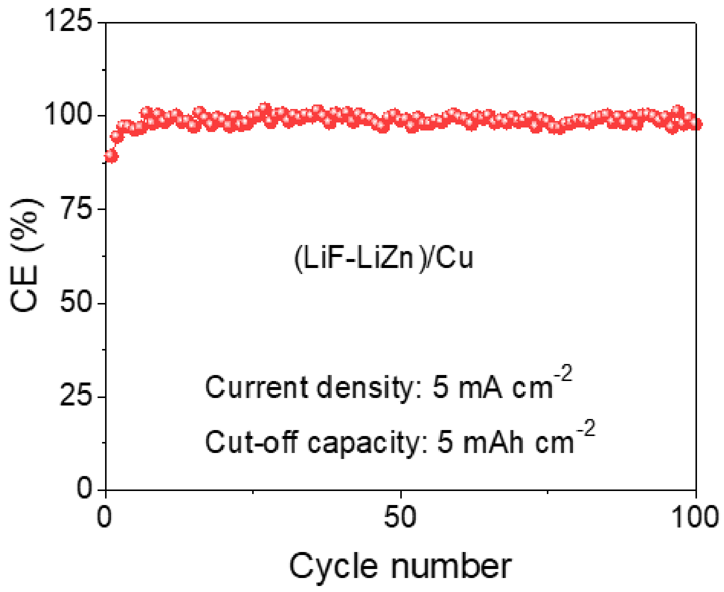

Figure S10. Coulombic efficiency of Li plating/stripping on (LiF-LiZn)/Cu at a current density of 5 mA cm^{-2} and cut-off capacity of 5 mAh cm^{-2}.

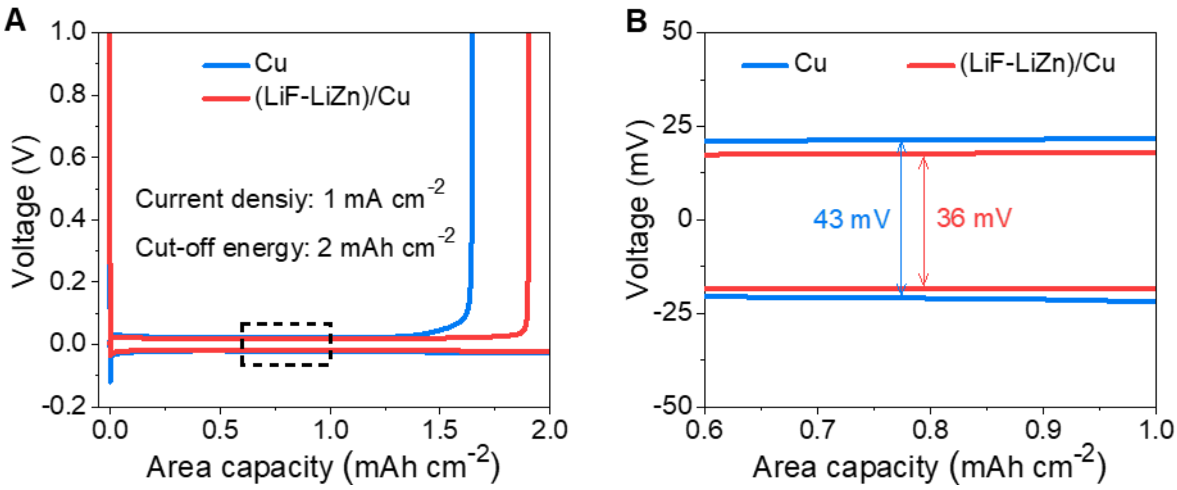

Figure S11. A) Voltage profiles of Li plating and stripping on Cu and (LiF-LiZn)/Cu CC at 1 mA cm^{-2}. B) Corresponding voltage hysteresis.

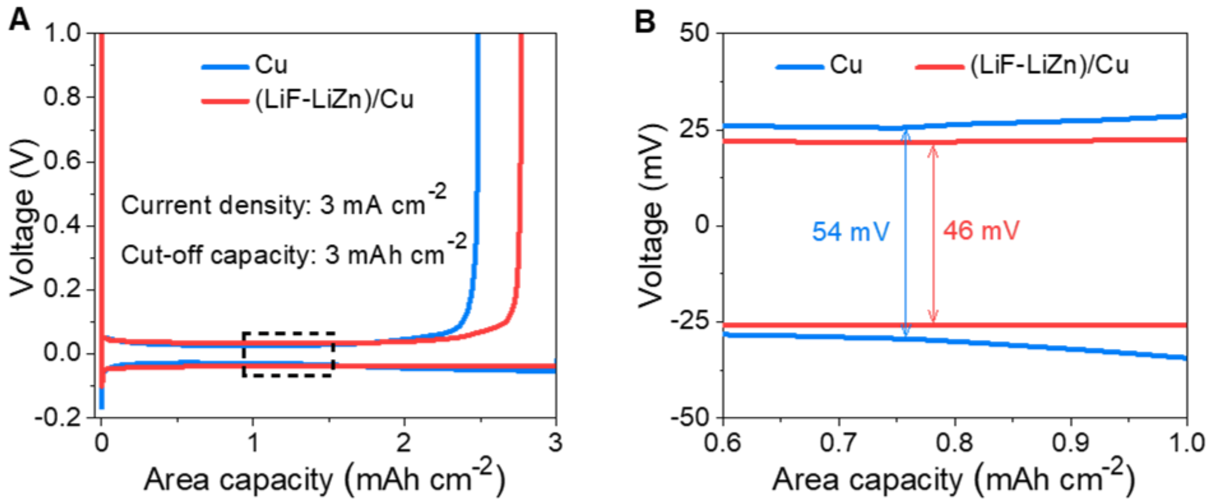

Figure S12. A) Voltage profiles of Li plating and stripping on Cu and (LiF-LiZn)/Cu CC at 3 mA cm^{-2}. B) Corresponding voltage hysteresis.

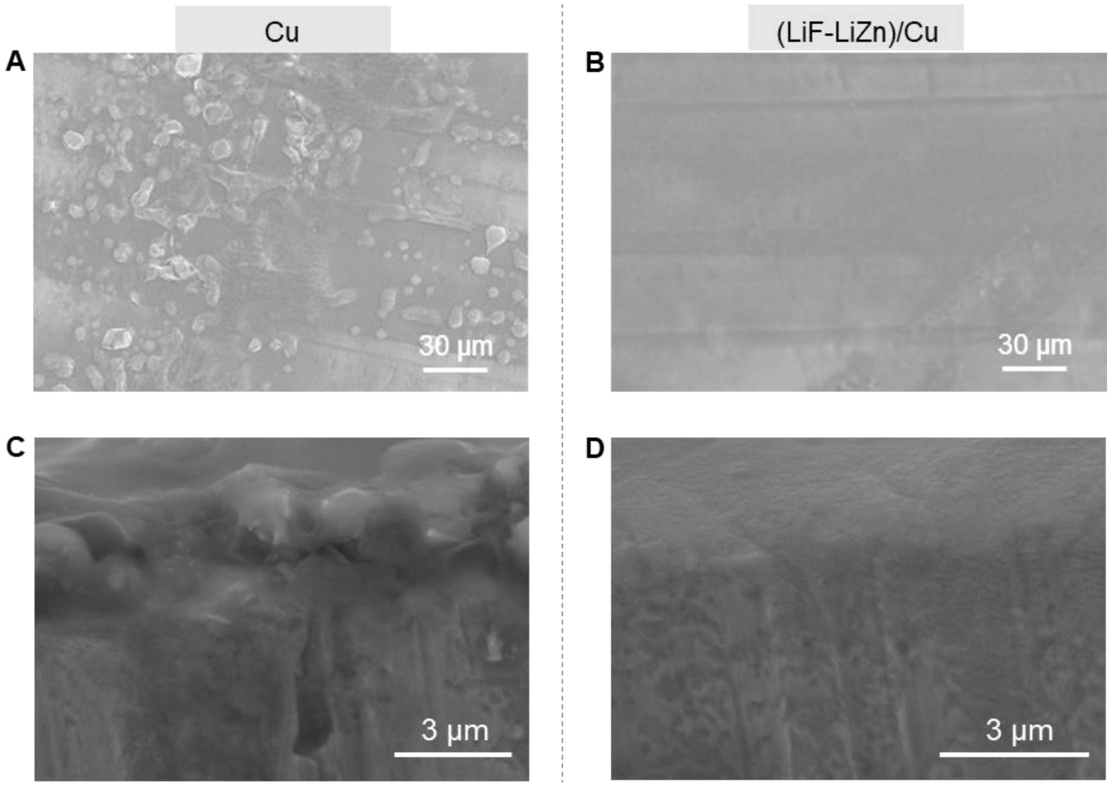

Figure S13. Morphologies of CCs after fully stripping Li. A–B) Top-view SEM images of Cu and (LiF-LiZn)/Cu, respectively. C–D) Cross-section SEM images of Cu and (LiF-LiZn)/Cu, respectively.

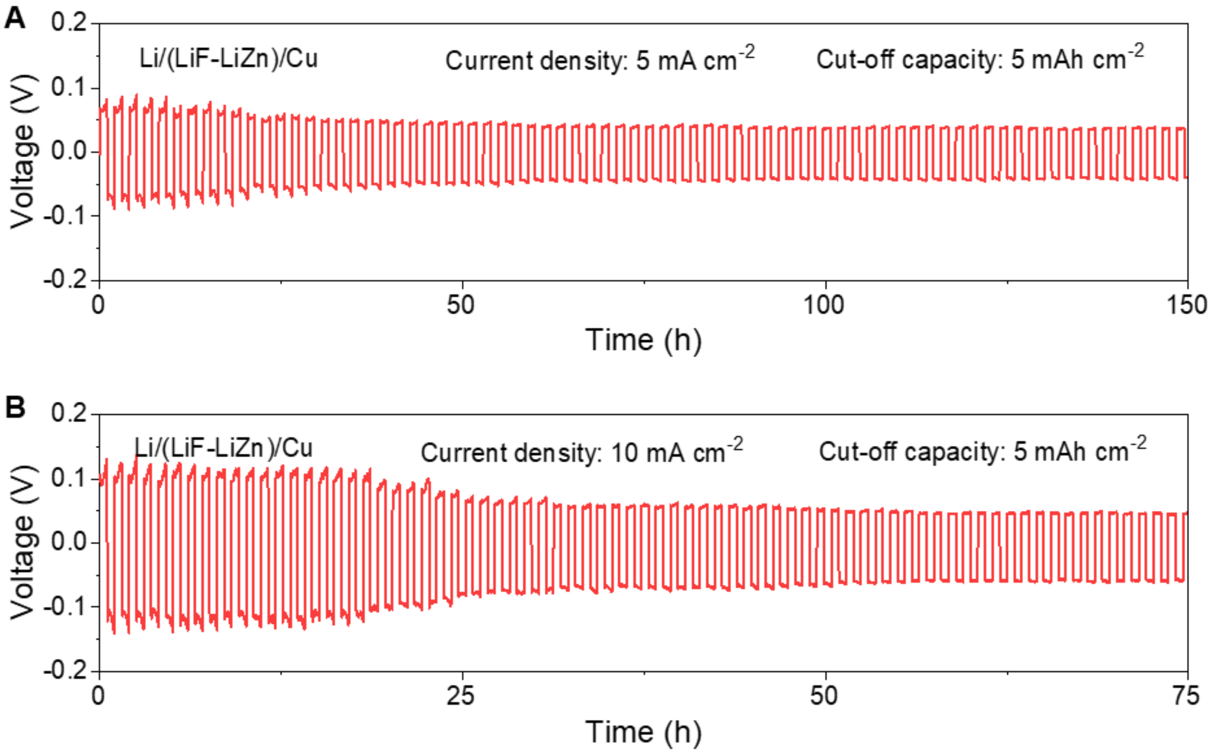

Figure S14. Cycling performance of symmetric cells using Li/(LiF-LiZn)/Cu. (A) At a current density of 5 mA cm^{-2} and cut-off capacity of 5 mAh cm^{-2}. (B) At a current density of 10 mA cm^{-2} and cut-off capacity of 5 mAh cm^{-2}.

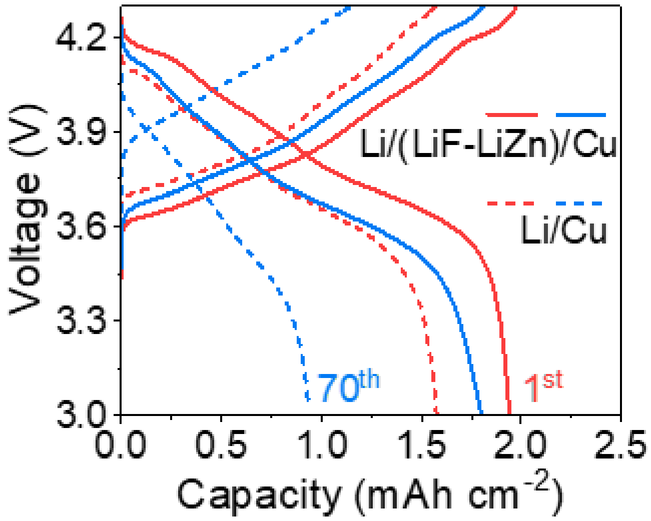

Figure S15. Discharge and charge voltage profiles of different Li‖NMC full cells with 6 mAh cm^{-2} of pre-deposited Li.

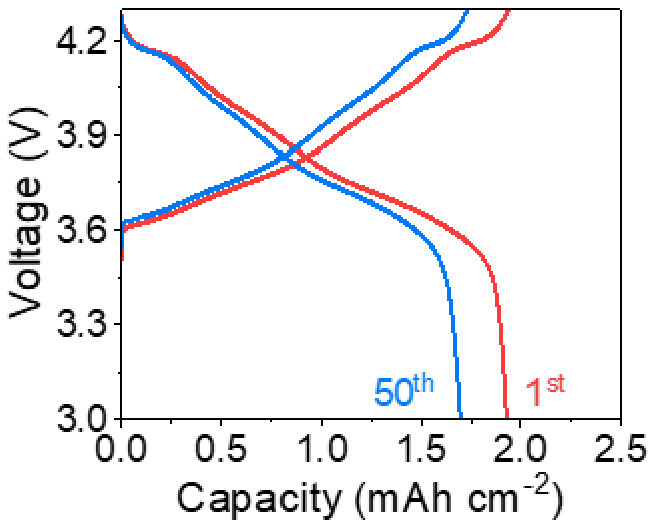

Figure S16. Discharge and charge voltage profiles of the Li/(LiF-LiZn)/Cu‖NMC "anode-less" cell with the N/P ratio of 0.9 at the 1st and 50th cycle.

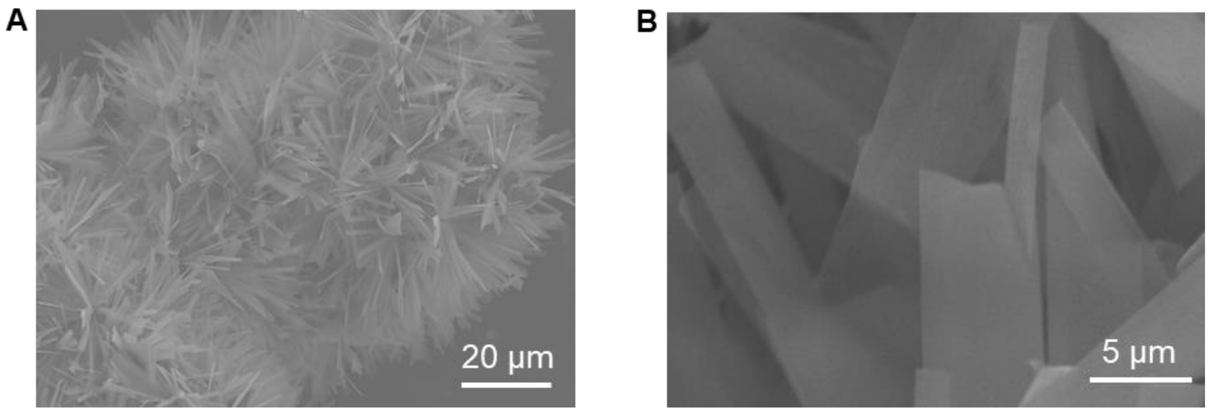

Figure S17. SEM images of TiO_2/TiS_2 at (A) low and (B) high resolutions.

Table S1. Comparison of the cycle time and voltage polarization between Li || Li symmetric cells utilizing Li/(LiF-LiZn)/Cu and previously reported LMB.

Electrode	Operating condition (mA cm^{-2}/ mAh cm^{-2})	Voltage polarization (mV)	Cycle time (h)	Ref.
**Cu HoMS**	1 / 1	~20	1200	S1
**LiF-rich Li**	1 / 1	~43	520	S2
**Li@G**	1 / 3	~27	400	S3
**LSSe@Li**	1.5 / 3	~17	900	S4
**Ag (Au)-Li**	1 / 1	~25	900	S5
**Cu@Zn-MOF/PVA**	1 / 1	~51.7	580	S6
**MXene/COF-LZU1**	1 / 1	~24	400	S7
**COF-Li**	0.5 / 1	~20	1000	S8
**P(St-Mal)@Li**	1 / 1	~20	900	S9
**Naked Li**	1 / 1	~30	1000	S10
**Li/(LiF-LiZn)/Cu**	**1 / 1**<br>**3 / 3**	**~17**<br>**~25**	**1000**<br>**900**	**This work**

3. References

(S1) Wei, P.; Wang, H.; Yang, M.; Wang, J.; Wang, D. Relocatable Hollow Multishelled Structure-Based Membrane Enables Dendrite-Free Lithium Deposition for Ultrastable Lithium Metal Batteries. *Adv. Energy Mater.* **2024**, 400108.

(S2) Yuan, Y.; Wu, F.; Bai, Y.; Li, Y.; Chen, G.; Wang, Z.; Wu, C. Regulating Li Deposition by Constructing LiF-Rich Host for Dendrite-Free Lithium Metal Anode. *Energy Storage Mater.* **2019**, *16*, 411–418.

(S3) He, B.; Deng, W.; Han, Q.; Zhu, W.; Hu, Z.; Fang, W.; Zhou, X.; Liu, Z. Scalable Fabrication of a Large-Area Lithium/Graphene Anode towards a Long-Life 350 W h Kg^{-1} Liithium Metal Pouch Cell. *J. Mater. Chem. A* **2021**, *9*, 25558–25566.

(S4) Liu, F.; Wang, L.; Zhang, Z.; Shi, P.; Feng, Y.; Yao, Y.; Ye, S.; Wang, H.; Wu, X.; Yu, Y. A Mixed Lithium-Ion Conductive Li_2S/Li_2Se Protection Layer for Stable Lithium Metal Anode. *Adv. Funct. Mater.* **2020**, *30*, 2001607.

(S5) Guo, F.; Wu, C.; Chen, H.; Zhong, F.; Ai, X.; Yang, H.; Qian, J. Dendrite-Free Lithium Deposition by Coating a Lithiophilic Heterogeneous Metal Layer on Lithium Metal Anode. *Energy Storage Mater.* **2020**, *24*, 635–643.

(S6) Fan, L.; Guo, Z.; Zhang, Y.; Wu, X.; Zhao, C.; Sun, X.; Yang, G.; Feng, Y.; Zhang, N. Stable Artificial Solid Electrolyte Interphase Films for Lithium Metal Anode: Via Metal-Organic Frameworks Cemented by Polyvinyl Alcohol. *J. Mater. Chem. A* **2020**, *8*, 251–258.

(S7) Wei, C.; Wang, Y.; Zhang, Y.; Tan, L.; Qian, Y.; Tao, Y.; Xiong, S.; Feng, J. Flexible and Stable 3D Lithium Metal Anodes Based on Self-Standing MXene/COF Frameworks for High-Performance Lithium–Sulfur Batteries. *Nano Res.* **2021**, *14*, 3576–3584.

(S8) Chen, D.; Huang, S.; Zhong, L.; Wang, S.; Xiao, M.; Han, D.; Meng, Y. In Situ Preparation of Thin and Rigid COF Film on Li Anode as Artificial Solid Electrolyte Interphase Layer Resisting Li Dendrite Puncture. *Adv. Funct. Mater.* **2020**, *30*, 1907717.

(S9) Naren, T.; Kuang, G. C.; Jiang, R.; Qing, P.; Yang, H.; Lin, J.; Chen, Y.; Wei, W.; Ji, X.; Chen, L. Reactive Polymer as Artificial Solid Electrolyte Interface for Stable Lithium Metal Batteries. *Angew. Chem. Int. Ed.* **2023**, *62*, 2305287.

(S10) Baek, M.; Kim, J.; Jeong, K.; Yang, S.; Kim, H.; Lee, J.; Kim, M.; Kim, K. J.; Choi, J. W. Naked Metallic Skin for Homo-Epitaxial Deposition in Lithium Metal Batteries. *Nat. Commun.* **2023**, *14*, 1296.
